# Body Mass Index, Physical Activity, and Fracture Among Young Adults: Longitudinal Results From the Thai Cohort Study

**DOI:** 10.2188/jea.JE20120215

**Published:** 2013-11-05

**Authors:** Susan Jordan, Lynette Lim, Janneke Berecki-Gisolf, Chris Bain, Sam-ang Seubsman, Adrian Sleigh, Emily Banks, the Thai Cohort Team

**Affiliations:** 1 School of Population Health, University of Queensland, Brisbane, Australia; 2 Population Health Department, The Queensland Institute of Medical Research, Brisbane, Australia; 3 National Centre for Epidemiology and Population Health, ANU College of Medicine, Biology and the Environment, The Australian National University, Canberra, Australia; 4 Monash Injury Research Institute, Monash University, Melbourne, Australia; 5 Thai Health-Risk Transition Study, School of Human Ecology, Sukhothai Thammathirat Open University, Nonthaburi, Thailand

**Keywords:** fracture, obesity, body size, physical activity, prevention, epidemiology, young adults

## Abstract

**Background:**

We investigated risk factors for fracture among young adults, particularly body mass index (BMI) and physical activity, which although associated with fracture in older populations have rarely been investigated in younger people.

**Methods:**

In 2009, 4 years after initial recruitment, 58 204 Thais aged 19 to 49 years were asked to self-report fractures incident in the preceding 4 years. Conditional logistic regression was used to calculate odds ratios (ORs) and 95% CIs for associations of fracture incidence with baseline BMI and physical activity.

**Results:**

Very obese women had a 70% increase in fracture risk (OR = 1.73, 95% CI 1.21–2.46) as compared with women with a normal BMI. Fracture risk increased by 15% with every 5-kg/m^2^ increase in BMI. The effects were strongest for fractures of the lower limbs. Frequent purposeful physical activity was also associated with increased fracture risk among women (OR = 1.52, 95% CI 1.12–2.06 for 15 episodes/week vs none). Neither BMI nor physical activity was associated with fracture among men, although fracture risk decreased by 4% with every additional 2 hours of average sitting time per day (OR = 0.96, 95% CI 0.93–0.99).

**Conclusions:**

The increase in obesity prevalence will likely increase fracture burden among young women but not young men. While active lifestyles have health benefits, our results highlight the importance of promoting injury prevention practices in conjunction with physical activity recommendations, particularly among women.

## INTRODUCTION

Fractures are an important cause of morbidity among young adults. They can result in prolonged absence from work, considerable health-resource use, and frequent long-term disability.^1^ They are particularly important in developing countries with young populations where injury accounts for a large proportion of the disease burden.^2^ Despite this, few studies have investigated risk factors for fracture among adults younger than 50 years. One research group has investigated the effects of dietary calcium,^3^ serum vitamin D,^4^ and levels of sex steroid hormones,^5^ but other exposures were not examined. Thus an evidence base for targeted prevention is lacking.

Low body mass index (BMI) and low physical activity level are 2 potentially modifiable factors that have been consistently associated with fracture risk among postmenopausal women and older men.^6^^,^^7^ However, it is unclear whether these factors have an effect on fracture risk among men and women during early and mid-adulthood.

Some studies of younger adults investigated the effects of BMI and physical activity on bone mineral density (BMD), a proxy for future fracture risk. Most found that BMD increases with weight^8^^–^^13^ and physical activity.^9^^,^^14^^,^^15^ However, this may not equate directly to fracture risk, as higher fat mass relative to lean body mass can adversely affect bone strength,^16^^–^^20^ fat mass may increase instability and propensity to fall,^21^ and the increased bone density associated with greater weight may not fully compensate for the extra load on bones.^22^ Furthermore, while physical activity may increase bone strength,^7^ it may also increase exposure to situations that increase fracture risk. Therefore it is important to examine fracture outcomes rather than proxies of such outcomes.

Obesity and physical inactivity are major health concerns in Western countries and emerging concerns in newly industrialized countries.^23^ We are therefore interested to see how BMI (as a measure of obesity) and physical activity influence actual fracture incidence among younger people. This will help us understand how increased BMI among younger people will affect the population burden of fracture and how uptake of physical activity recommendations could modify this relationship. We have used longitudinal data from a large study of young Thai adults to investigate these relationships in detail.

## METHODS

The Thai Cohort Study was established to examine the health consequences of Thailand’s rapid socioeconomic development. The methods have been reported previously.^24^ In brief, participants were recruited from the student body of Sukhothai Thammathirat Open University (STOU). STOU students reside all over Thailand and have a modest socioeconomic status. Many are rural dwellers and most work full-time. In 2005 all STOU students who had completed at least 1 semester of study (approximately 200 000) were invited to participate. Overall, 87 134 students (47 314 women and 39 820 men) completed questionnaires (44%).

Baseline measures included demographic variables (age, sex, area of residence, income, and prior education), lifestyle variables (smoking, alcohol, and physical activity), medical diagnoses (diabetes, ischemic heart disease, kidney disease, thyroid disease, and cancer) and self-reported height and weight. We calculated BMI as weight (kg)/height (m)^2^ and categorized this using suggested cut-points for overweight and obesity among Asian populations.^25^ We also assessed the effect of height on fracture risk because some studies have suggested that height could be associated with some fracture types.^26^^–^^28^ The number of episodes of physical activity per week was calculated by summing responses to questions on number of episodes of walking (at work, home, or for exercise), plus episodes of mild (eg, Tai Chi), moderate (eg, cycling at a regular pace, carrying light loads), and strenuous (eg, heavy lifting, digging, running) exercise. Participants were not asked to exclude exercise that was done during their work. We also considered separately the effect of frequency of housework/gardening (seldom/never; 1–3 times/month; 1–2 times/week; 3–4 times/week; and most days), as this provided discriminating estimates of physical activity in previous analyses.^29^ Sitting time was ascertained by the question, “How many hours per day do you usually spend sitting for any purpose (eg, reading, resting, working, thinking)?”.

In 2009, a 4-year follow-up mail survey was conducted. Of the initial 87 134 participants, 289 died before the follow-up survey, 20 withdrew from the study, and an additional 1608 had no contact details or insufficient information on their identity. A follow-up survey was mailed to 85 217. After 4 rounds of mail-outs and more than 80 000 telephone calls, there were 10 207 for whom we still could not confirm an address. A further 14 441 did not return their survey, and 60 569 participants replied (71.1% of the 85 217 approached). Of these, 55% were women. Median age was 34 years (range 19–92).

Using the question, “In your life have you ever experienced a fracture to the areas of your body mentioned below?”, participants were asked to indicate whether they had experienced a fracture at any of 13 specified sites (finger/toe; wrist; arm; collarbone; rib; skull; face/jaw/nose; neck; back; pelvis; leg; ankle; other) during their lifetime and the age at which they sustained the fracture. Fractures reported to have occurred at an age greater than the participant’s age at baseline were considered to be incident fractures. Variables related to women’s contraceptive use were also taken from the 2009 survey.

### Statistical analyses

Men and women were considered separately in the analyses, due to the possibility that fracture etiology might vary by sex. We decided a priori to exclude adults older than 50 years because our interest was the effect of BMI and physical activity on younger adults. Furthermore, very few of our participants (approximately 3%) were in that older age group and, while we considered the possibility that associations might vary by menopausal status, we lacked the statistical power to test for such an interaction. In addition we excluded those with a cancer diagnosis (*n* = 371) because we felt that adjusting for this might not adequately account for the broad spectrum of effects of different cancers on BMI and fracture occurrence. Skull fractures may have been over-reported (due to confusion with head trauma more generally), so these were also excluded, as were events for which fracture information was missing (*n* = 21). In site-specific analyses we grouped wrist and arm fractures (upper limb) and leg and ankle fractures (lower limb).

Participants were asked to record the age at which their fracture(s) was sustained rather than the date, because age at fracture is far more likely to be recalled than the date of a fracture. Thus our information was not precise enough to undertake a survival-type analysis. Univariate analyses stratified by age group (10-year bands) suggested that associations between fracture and BMI/physical activity persisted across all ages, but we were concerned that adjusting for age in our analyses might not be sufficient given the strong relationships of increasing age with both BMI and fracture. As a result we have undertaken all multivariable analyses using conditional logistic regression, stratified by age in years, to calculate odds ratios (ORs) and 95% CIs as estimates of relative risk. All analyses were adjusted for history of fracture before 2005 (yes/no), income (≤7000, >7000–10 000, >10 000–20 000, >20 000 Thai Baht), smoking status (ever/never), ever-use of alcohol (yes/no), vascular disease (ischemic heart disease or stroke; yes/no). Analyses considering BMI were adjusted for episodes of purposeful physical activity per week (0, 1–5, 6–10, 11–15, >15), and analyses considering physical activity were adjusted for BMI (<18.5, 18.5 to <23, 23 to <24.5, 25 to <30, ≥30.0 kg/m^2^). For women, analyses were additionally adjusted for ever-use of depot medroxyprogesterone acetate (DMPA). Factors such as parity, prior education, and place of residence, which did not materially change the estimates, were excluded from the final model. To assess trends of risk with increasing exposure, the continuous forms of the variables divided by the interval of interest (ie, 10 cm for height; 5 kg/m^2^ for BMI; per 5 episodes for physical activity; per 2 hours of sitting time) were used in the models. For amount of housework/gardening the categorical variable was entered into the model as a linear term and its significance assessed. All analyses were done using SAS statistical software (version 9.2, SAS Institute Inc., Cary, NC, USA).

Ethics approval was obtained from the STOU Research and Development Institute (protocol 0522/10) and the Australian National University Human Research Ethics Committee (protocol 2004344). Informed written consent was obtained from all participants.

## RESULTS

Our analyses included 32 339 women and 25 865 men younger than 50 who completed the survey in 2009. Of these, 902 (2.8%) women and 1168 (4.5%) men reported having had a fracture since 2005. Participant characteristics are summarized in Table 1. Fracture was more common among older women than among younger women, whereas the opposite trend was seen among men (*P* < 0.0001 respectively). For both sexes those who smoked or had vascular disease or a fracture before 2005 were significantly more likely to have had a fracture since 2005. Significant associations were also seen for BMI and frequency of physical activity. For women an association was also seen for ever use of DMPA contraceptives; while for men, income, prior education, and ever-use of alcohol were also significantly associated with fracture incidence.

**Table 1.** Baseline characteristics of women and men in the Thai Cohort Study (2005–2009), according to self-report of fracture since 2005

	Women - Fracture			Men - Fracture		
		
	Yes (*n* = 902)	No (*n* = 31 437)	*P*-value^a^	Yes (*n* = 1168)	No (*n* = 24 697)	*P*-value^a^
	Mean (SD)			Mean (SD)		
		
Age (yrs)	30.9 (7.5)	29.7 (7.1)	<0.0001	31.5 (7.3)	32.5 (7.6)	<0.0001
Height (cm)	158.0 (5.8)	157.5 (5.5)	0.01	168.7 (5.9)	168.3 (5.9)	0.02
	*n*^b^ (%)			*n*^b^ (%)		
		
Income (Thai baht)						
≤7000	366 (41.5)	13 664 (44.5)	0.3	389 (33.7)	7759 (32.1)	<0.0001
>7000–10 000	218 (24.7)	7419 (24.2)		323 (28.0)	5660 (23.4)	
>10 000–20 000	222 (25.2)	7020 (22.9)		319 (27.6)	7560 (31.2)	
>20 000	76 (8.6)	2623 (8.5)		123 (10.7)	3229 (13.3)	
Prior education						
Junior high	22 (2.5)	603 (2.1)	0.1	46 (4.0)	1110 (4.5)	0.03
High school	378 (42.1)	12 173 (37.7)		588 (50.4)	11 885 (47.8)	
Diploma/certificate	253 (28.1)	9632 (30.7)		288 (24.7)	5658 (23.0)	
University	246 (27.4)	8953 (28.6)		244 (20.9)	5993 (24.3)	
Place of residence						
City	487 (54.4)	16 221 (51.9)	0.2	596 (51.2)	12 121 (49.4)	0.7
Countryside	409 (45.7)	15 011 (48.1)		568 (48.8)	12 413 (50.6)	
Parity						
0	543 (61.8)	20 164 (65.6)	0.07			
1	166 (18.9)	5216 (17.0)				
2	135 (15.4)	4449 (14.1)				
≥3	35 (4.0)	923 (3.0)				
Body mass index						
<18.5	156 (17.3)	6434 (20.5)	<0.0001	57 (4.9)	1407 (5.7)	0.04
18.5–<23.0	528 (58.4)	18 423 (58.6)		610 (52.2)	11 758 (47.6)	
23.0–<25.0	85 (9.4)	3127 (10.0)		239 (20.5)	5443 (22.0)	
25.0–<30.0	88 (9.8)	2473 (7.9)		220 (18.8)	4972 (20.1)	
≥30.00	36 (4.0)	641 (2.0)		28 (2.4)	754 (3.1)	
Physical activity (episodes/week)						
0	72 (8.0)	3028 (9.6)	0.002	58 (5.0)	1429 (5.8)	0.002
1–5	228 (25.3)	9329 (29.7)		248 (21.2)	5473 (22.2)	
6–10	323 (35.8)	10 743 (34.2)		327 (28.0)	7567 (30.7)	
11–15	144 (16.0)	4416 (14.1)		285 (24.4)	4786 (19.4)	
>15	117 (13.0)	3250 (10.3)		232 (19.9)	4968 (20.1)	
Missing	18 (2.0)	669 (2.1)		18 (1.5)	469 (1.9)	
Smoking						
Ever	67 (7.7)	1587 (5.2)	0.001	642 (56.3)	12 669 (52.4)	0.01
Never	805 (92.3)	28 947 (94.8)		499 (43.7)	11 525 (47.6)	
Alcohol consumption						
Current	468 (52.5)	16 283 (52.5)	0.9	945 (81.6)	19 280 (78.8)	0.02
None	423 (47.5)	14 727 (47.5)		213 (18.4)	5182 (21.2)	
Ever used DMPA^c^						
Yes	169 (18.7)	4733 (15.1)	0.002			
No	733 (81.3)	26 690 (84.9)				
Thyroid disease						
Yes	45 (5.0)	1666 (5.3)	0.7	20 (1.7)	336 (1.4)	0.3
No	857 (95.0)	29 771 (94.7)		1148 (98.3)	24 360 (98.6)	
Kidney disease						
Yes	32 (3.6)	796 (2.5)	0.06	38 (3.3)	605 (2.5)	0.08
No	870 (96.5)	30 641 (97.5)		1130 (96.8)	24 091 (97.6)	
Vascular disease						
Yes	10 (1.1)	134 (0.4)	0.002	13 (1.1)	139 (0.6)	0.02
No	892 (98.9)	31 303 (99.6)		1155 (98.9)	24 557 (99.4)	
Fracture before 2005						
Yes	201 (22.3)	4282 (13.6)	<0.0001	427 (36.6)	7088 (28.7)	<0.0001
No	701 (77.7)	27 155 (86.4)		741 (63.4)	17 609 (71.3)	

^a^*P*-values are from *t*-test for continuous variables and χ^2^ tests for categorical variables. *P* < 0.05 is considered significant.

^b^Numbers may not sum to total because of missing data.

^c^DMPA = depot medroxyprogesterone acetate.

Table 2 shows the results of multivariable analyses of the associations of fracture incidence with BMI and height. Women in the highest category of obesity had a 70% higher risk of fracture as compared with those with normal BMI values (OR 1.73, 95% CI 1.21–2.46), and risk increased by 15% for every additional 5-kg/m^2^ increase in BMI. Being underweight did not increase fracture risk. For men there was no linear association between BMI and fracture after adjustment for age, although underweight men had a significant 26% decrease in risk as compared with men in the normal BMI range. The interaction term for sex and BMI was significant (*P* = 0.002). There was a suggestion that taller men and taller women had an increased fracture risk as compared with shorter participants, but the effect was not statistically significant.

**Table 2.** Associations of body mass index (BMI) and height with fracture incidence among 32 339 women and 25 865 men in the Thai Cohort Study (2005–2009)

	Women				Men			
		
	Total *n*^a^	Fracture *n* (%)	OR 95%CI^b^	OR 95%CI^c,d^	Total *n*^a^	Fracture *n* (%)	OR 95%CI^b^	OR 95%CI^c^
BMI								
<18.5	6590	156 (2.4)	0.93 (0.77–1.12)	0.94 (0.78–1.14)	1464	57 (3.9)	0.72 (0.54–0.96)	0.74 (0.56–0.99)
18.5–<23.0	18 951	528 (2.8)	1.00	1.00	12 368	610 (4.9)	1.00	1.00
23.0–<25.0	3212	85 (2.7)	0.91 (0.71–1.15)	0.90 (0.71–1.14)	5682	239 (4.2)	0.92 (0.79–1.07)	0.92 (0.79–1.07)
25.0–<30.0	2561	88 (3.4)	1.16 (0.91–1.48)	1.11 (0.87–1.41)	5192	220 (4.2)	0.94 (0.80–1.10)	0.93 (0.79–1.09)
≥30.00	677	36 (5.3)	1.82 (1.27–2.62)	1.73 (1.21–2.46)	782	28 (3.6)	0.76 (0.52–1.12)	0.77 (0.52–1.13)
Per 5-kg/m^2^ increase			1.17 (1.06–1.30)	1.15 (1.04–1.27)			0.99 (0.90–1.09)	0.98 (0.89–1.08)
Height per 10 cm^e^			1.23 (1.09–1.39)	1.12 (0.98–1.28)			1.09 (0.99–1.21)	1.10 (0.98–1.24)

^a^Numbers may not sum to total because of missing data.

^b^From univariate conditional logistic regression model stratified by age.

^c^Adjusted for fracture before 2005 (yes/no), income (≤7000, >7000–10 000, >10 000–20 000, >20 000 Baht), smoking status (ever/ never), ever-use of alcohol (yes/no), vascular disease (ischemic heart disease or stroke – yes/no), and episodes of purposeful physical activity per week (0, 1–5, 6–10, 11–15, >15).

^d^Additionally adjusted for ever-use of depot medroxyprogesterone acetate.

^e^Additionally adjusted for self-reported weight in kilograms.

Associations between physical activity measures and fracture risk are shown in Table 3. Among women fracture risk increased with increasing episodes of purposeful physical activity (excluding gardening and housework) (OR = 1.52, 95% CI 1.12–2.06 for >15 episodes per week vs none), but no significant relationship was observed among men (*P*(interaction) = 0.06). Conversely, more sitting time (adjusted for episodes of physical activity) modestly decreased fracture risk among men (OR 0.96, 95% CI 0.93–0.99 per additional 2 hours of sitting time per day) but not women, although the interaction term was not significant (*P* = 0.4). There was no significant association of frequency of household chores or manual worker status (versus those who reported being professionals, office workers, or managers) with fracture.

**Table 3.** Association between measures of physical activity and fracture incidence among 32 339 women and 25 865 men in the Thai Cohort Study (2005–2009)

	Women				Men			
		
	Total *n*^a^	Fracture *n* (%)	OR 95%CI^b^	OR 95%CI^c,d^	Total *n*^a^	Fracture *n* (%)	OR 95%CI^b^	OR 95%CI^c^
Purposeful physical activity (episodes per week)								
0	3100	72 (2.3)	1.00	1.00	1488	58 (3.9)	1.00	1.00
1–5	9558	228 (2.4)	1.04 (0.79–1.36)	1.05 (0.80–1.38)	5722	248 (4.3)	0.90 (0.67–1.21)	1.12 (0.83–1.48)
6–10	11 066	323 (2.9)	1.26 (0.97–1.64)	1.28 (0.98–1.67)	7896	327 (4.1)	1.01 (0.84–1.21)	1.02 (0.77–1.35)
11–15	4561	144 (3.2)	1.33 (0.99–1.79)	1.34 (1.00–1.80)	5072	285 (5.6)	0.94 (0.79–1.11)	1.37 (1.02–1.82)
>15	3367	117 (3.5)	1.52 (1.12–2.05)	1.52 (1.12–2.06)	5201	232 (4.5)	1.26 (1.06–1.51)	1.08 (0.80–1.45)
			*P*_trend_ = 0.002	*P*_trend_ = 0.004			*P*_trend_ = 0.3	*P*_trend_ = 0.4
Per 5 episodes/week			1.05 (1.02–1.08)	1.04 (1.01–1.08)			1.01 (0.99–1.04)	1.01 (0.99–1.04)
Housework/gardening								
Seldom/never	1106	24 (2.2)	1.00	1.00	2114	96 (4.5)	1.00	1.00
1–3/month	2468	70 (2.8)	1.28 (0.80–2.06)	1.22 (0.76–1.96)	3827	178 (4.7)	0.98 (0.76–1.26)	0.96 (0.74–1.24)
1–2/week	8636	233 (2.7)	1.23 (0.80–1.89)	1.17 (0.76–1.80)	7142	348 (4.9)	1.03 (0.82–1.29)	0.99 (0.78–1.25)
3–4/week	3802	85 (2.2)	1.06 (0.67–1.68)	0.98 (0.61–1.56)	3548	154 (4.3)	0.92 (0.71–1.19)	0.86 (0.66–1.12)
Most days	15 968	479 (3.0)	1.33 (0.87–2.02)	1.22 (0.80–1.87)	8850	376 (4.3)	0.93 (0.74–1.17)	0.87 (0.69–1.10)
			*P*_trend_ = 0.2	*P*_trend_ = 0.5			*P*_trend_ = 0.3	*P*_trend_ = 0.09
Manual worker^e^								
No	22 896	634 (2.8)	1.00	1.00	18 160	794 (4.4)	1.00	1.00
Yes	5355	167 (3.1)	1.16 (0.97–1.39)	1.11 (0.92–1.33)	4969	258 (5.2)	1.15 (0.99–1.32)	1.10 (0.95–1.28)
Sitting time (hrs/day)^e^								
0–3	8023	231 (2.9)	1.00	1.00	7367	341 (4.6)	1.00	1.00
4–7	8953	248 (2.8)	0.97 (0.80–1.17)	0.96 (0.80–1.16)	8683	418 (4.8)	1.04 (0.90–1.20)	1.04 (0.90–1.20)
8–9	5965	172 (2.9)	1.03 (0.84–1.26)	1.04 (0.84–1.27)	3816	169 (4.3)	0.96 (0.79–1.15)	0.96 (0.79–1.16)
≥10	8949	243 (2.7)	0.99 (0.82–1.19)	0.99 (0.82–1.20)	5624	223 (4.0)	0.84 (0.70–0.99)	0.84 (0.71–1.00)
Per 2 hours			1.00 (0.97–1.04)	1.00 (0.97–1.04)			0.96 (0.93–0.99)	0.96 (0.93–0.99)

^a^Numbers may not sum to total because of missing data.

^b^From univariate conditional logistic regression model stratified by age.

^c^Adjusted for fracture before 2005 (yes/no), income (≤7000, >7000–10 000, >10 000–20 000, >20 000 Baht), smoking status (ever/never), ever-use of alcohol (yes/no), vascular disease (ischemic heart disease or stroke – yes/no), and body mass index (<18.5, 18.5–<23, 23–<24.5, 25–<30, ≥30.0 kg/m^2^).

^d^Additionally adjusted for ever use of depot medroxyprogesterone acetate.

^e^Multivariable models additionally adjusted for episodes of purposeful physical activity per week (0, 1–5, 6–10, 11–15, >15).

We stratified our analyses to test if the association between BMI and fracture among women was modified by the frequency of physical activity (in categories of frequency) but found no evidence of such an effect (*P*(interaction) = 0.7).

Table 4 shows the effects of BMI, number of episodes of purposeful physical activity, and hours of sitting time on site-specific fracture risk among women and men. Among women, obesity was most strongly associated with lower limb fracture (ankle and leg), with a significant 32% increase in fracture risk per 5-unit increase in BMI. The risk of upper limb fracture was nonsignificantly elevated among very obese women. There were no clear patterns of association between BMI and site-specific fracture risk among men. There was also a suggestion that more-frequent purposeful physical activity modestly increased fracture risk at all sites among women, although the estimates were not statistically significant for upper or lower limb fractures. Purposeful physical activity was not clearly associated with fracture at any site among men. As for the main results, each additional 2 hours of sitting time per day reduced the risk of lower limb fracture by 6% (OR 0.94, 95% CI 0.88–0.99) among men.

**Table 4.** Association of body mass index (BMI) and measures of physical activity with site-specific fracture incidence among women and men in the Thai Cohort Study

	Women			Men		
		
	Upper limb (*n* = 137)^a^	Lower limb (*n* = 240)^a^	All other sites (*n* = 535)^a^	Upper limb (*n* = 192)^a^	Lower limb (*n* = 311)^a^	All other sites (*n* = 694)^a^
	OR 95%CI^b,c^			OR 95%CI^b^		
BMI (kg/m^2^)						
<18.5	0.76 (0.46–1.24)	0.89 (0.61–1.30)	1.00 (0.79–1.27)	0.44 (0.19–1.00)	0.75 (0.44–1.29)	0.80 (0.55–1.16)
18.5–<23.0	1.00	1.00	1.00	1.00	1.00	1.00
23.0–<25.0	0.59 (0.30–1.19)	1.13 (0.73–1.74)	0.89 (0.65–1.21)	0.86 (0.59–1.25)	0.74 (0.54–1.02)	1.02 (0.84–1.24)
25.0–30.0	0.49 (0.21–1.13)	1.40 (0.90–2.15)	1.13 (0.83–1.54)	0.77 (0.51–1.18)	0.97 (0.71–1.31)	0.96 (0.77–1.18)
≥30.00	1.50 (0.60–3.72)	2.35 (1.26–4.37)	1.50 (0.92–2.45)	0.65 (0.24–1.77)	0.71 (0.33–1.52)	0.79 (0.48–1.30)
Per 5-kg/m^2^ increase	1.01 (0.77–1.34)	1.32 (1.10–1.57)	1.10 (0.96–1.26)	0.95 (0.75–1.20)	0.98 (0.82–1.18)	0.99 (0.87–1.12)
Purposeful physical activity (episodes per week)						
0	1.00	1.00	1.00	1.00	1.00	1.00
1–5	1.18 (0.56–2.47)	0.79 (0.48–1.32)	1.16 (0.81–1.67)	0.74 (0.39–1.39)	1.94 (0.97–3.90)	1.04 (0.72–1.51)
6–10	1.21 (0.59–2.52)	1.08 (0.69–1.79)	1.38 (0.97–1.96)	0.66 (0.36–1.22)	1.57 (0.79–3.13)	1.03 (0.72–1.47)
11–15	1.92 (0.90–4.13)	1.14 (0.67–1.96)	1.29 (0.87–1.91)	0.72 (0.38–1.37)	2.67 (1.34–5.31)	1.27 (0.88–1.84)
>15	1.97 (0.89–4.36)	1.26 (0.72–2.21)	1.57 (1.05–2.35)	0.85 (0.46–1.59)	1.69 (0.84–3.43)	1.02 (0.70–1.49)
	*P*_trend_ = 0.1	*P*_trend_ = 0.2	*P*_trend_ = 0.03	*P*_trend_ = 0.9	*P*_trend_ = 0.6	*P*_trend_ = 0.4
Sitting time (hrs/day)						
0–3	1.00	1.00	1.00	1.00	1.00	1.00
4–7	0.89 (0.55–1.44)	0.91 (0.64–1.29)	1.01 (0.79–1.30)	1.07 (0.74–1.55)	0.99 (0.75–1.31)	1.11 (0.92–1.34)
8–9	0.95 (0.56–1.63)	0.89 (0.60–1.32)	1.13 (0.86–1.48)	1.33 (0.87–2.07)	0.84 (0.58–1.21)	0.94 (0.73–1.20)
≥10	0.91 (0.56–1.48)	0.83 (0.58–1.20)	1.10 (0.86–1.40)	0.92 (0.60–1.42)	0.76 (0.55–1.07)	0.85 (0.68–1.07)
Per 2-hour increase	1.00 (0.92–1.09)	0.98 (0.92–1.05)	1.02 (0.98–1.07)	0.98 (0.91–1.06)	0.94 (0.88–0.99)	0.97 (0.93–1.01)

^a^Denotes number of incident fractures reported at this site.

^b^Adjusted for fracture before 2005 (yes/no), income (≤7000, >7000–10 000, >10 000–20 000, >20 000 Baht), smoking status (ever/ never), ever-use of alcohol (yes/no), vascular disease (ischemic heart disease or stroke – yes/no), kidney disease (yes/no), and episodes of purposeful physical activity per week (0, 1–5, 6–10, 11–15, >15), and BMI (<18.5, 18.5–<23, 23–<24.5, 25–<30, ≥30.0 kg/m^2^).

^c^Additionally adjusted for ever-use of depot medroxyprogesterone acetate (women only).

## DISCUSSION

In summary, in this cohort of young and middle-aged Thai adults, the risk of fracture, particularly lower limb fracture, increased with increasing BMI among women. Underweight men appeared to have a lower fracture risk as compared with men with higher BMI values. Among women, frequent purposeful physical activity modestly increased fracture risk at all sites, but gardening/housework did not. Among men, greater sitting time was inversely associated with fracture risk independent of purposeful physical activity.

The strengths of this study include the large sample size and prospective nature of the data, although all information, including fracture outcomes, was self-reported. Self-report is a relatively accurate way to obtain information on fracture incidence,^30^ although accuracy varies somewhat by site: arm and leg fractures are well reported but fractures of the hands, feet, and ribs are both under- and over-reported.^30^^,^^31^ Fractures that occurred further in the past may have been under-reported, which may have attenuated risk estimates,^32^ although repeating the analyses after including only fractures reported for the year before ascertainment did not materially alter the estimates. Over-reporting of height and under-reporting of weight were found to lead to modest BMI misclassification within the Thai Cohort Study, particularly in the overweight and obese categories,^33^ which could attenuate the association between BMI and fracture risk.

It is possible that the 29% loss to follow-up may have affected our findings. Those who did not complete the second survey were more likely to be single, male, younger, have less prior education, and to have been current smokers or drinkers at baseline, all of which were associated with fracture in our analyses. Physical activity was not related to attrition but obesity was (32% of women in the obese II category at baseline did not complete the second survey as compared with 28% of women in the normal BMI range). However, because reported injury in 2005 was not associated with attrition, there is little reason to expect fractures to be related to participation and, as the models were adjusted for all baseline variables related with attrition, it is unlikely that attrition has materially affected our results.^34^

Participants in the study were a relatively well-educated, ethnically homogeneous group of Thai people, which raises issues of generalizability. Although it is unlikely that the relationship between obesity and fracture varies by ethnicity, the circumstances of fracture occurrence might vary across countries. It may be that mode of fracture (eg, traffic accident vs fall, trauma vs fragility) affected the observed associations; however, we had no information on how fractures occurred. We plan to explore this issue further in subsequent data collections.

BMI and physical activity can positively and negatively influence fracture risk. The overall effect of BMI and physical activity on fracture will therefore be determined by the balance of these factors in an individual. In this cohort, fracture risk was not increased in underweight participants, suggesting that if, on average, underweight reduces bone mineral density in young people in this population, this does not result in more broken bones. Fracture risk was increased in very obese women. Perhaps this increase was mediated by an increased likelihood of trauma (falls) and its consequences, and/or the negative effects of severe obesity on bone strength.^20^ There are few data on fracture among younger adults, but in peri-/post-menopausal women higher BMI probably increases risk of ankle fracture but decreases risk of wrist and hip fracture.^35^ Our study had limited power to investigate site-specific effects, but in this young cohort upper limb fracture risk was not increased in underweight women. However, lower limb (leg and ankle) fracture was most strongly associated with high BMI, in keeping with findings in peri-/post-menopausal women.^26^^,^^35^^,^^36^ It has been suggested that this association is due to (a) the fact that greater body weight increases forces on the leg and ankle during a fall and/or (b) the increased propensity to fall among obese people, because of greater instability.^26^^,^^36^

Among women we found a modest increase in overall fracture incidence with increasing frequency of purposeful physical activity (ie, exercise), although household activity was not associated with risk. This suggests that the physical circumstances in which the activity was done, rather than the exercise per se, contributed to fracture risk in this population. Notably, we found no evidence that any of our physical activity measures were associated with decreased fracture risk among women, suggesting that, even if physical activity increases bone mineral density in this population, it is not of general relevance to fracture risk in this age group. However, our measures did not clearly distinguish between low- and high-impact physical activity and may thus not have captured important discriminating information.^37^

The effects of BMI and physical activity on fracture risk varied between men and women in this population. It is possible that relative differences in fat versus muscle mass partly explain this difference because women tend to have a higher percentage of body fat as compared with men with the same BMI,^38^ and higher fat mass relative to lean body mass can adversely affect bone strength^16^^–^^20^ and may increase instability and propensity to fall.^21^ It is also likely that the circumstances in which fractures are sustained vary substantially between sexes. Although we have not explored other factors in depth, we noted that socioeconomic indicators, younger age, and alcohol consumption were significantly associated with fracture occurrence among men, suggesting that risk behaviors may be more important drivers of fracture risk in younger men. Furthermore, the inverse association with sitting time in men may indicate that men who spend more time sitting are less likely to be in physical situations where fracture occurs. These differences warrant further investigation.

In conclusion, our results suggest that, among young women, fracture risk increases with increasing BMI and with frequent purposeful physical activity. The growing prevalence of obesity is therefore likely to increase the overall fracture burden in young women but not in men. Although an active lifestyle has obvious health benefits, particularly for those who are obese, our results highlight the importance of promoting injury prevention practices alongside physical activity recommendations.
